# Antibiotic-impregnated bone graft to prevent infection after total hip arthroplasty (ABOGRAFT): protocol for a randomised, double-blind, placebo-controlled trial

**DOI:** 10.1136/bmjopen-2025-114161

**Published:** 2026-09-11

**Authors:** Daphne Wezenberg, Nils P Hailer, Olof Sköldenberg, Anna Stefánsdóttir, Georgios Tsikandylakis, Peter Wildeman, Johan Kärrholm, Bo Söderquist, Lennart E Nilsson, Jörg Schilcher

**Affiliations:** 1 Department of Biomedical and Clinical Sciences, Linköping University, Linköping, Sweden; 2 Clinical Department of Orthopaedics, Region Östergötland, Linköping, Sweden; 3 Department of Surgical Sciences/Orthopedics, Uppsala University, Uppsala, Sweden; 4 Department of Clinical Sciences, Danderyd Hospital, Division of Orthopedics, Karolinska Institute, Stockholm, Sweden; 5 Department of Clinical Sciences, Division of Orthopedics, Department of Orthopedics, Lund University, Skåne University Hospital, Lund, Sweden; 6 Department of Orthopedics, Institute of Clinical Sciences, Sahlgrenska Academy, University of Gothenburg, Gothenburg, Sweden; 7 Department of Orthopedics, Faculty of Medicine and Health, Örebro Universitet, Örebro, Sweden; 8 School of Medical Sciences, Faculty of Medicine and Health, Örebro University, Örebro, Sweden; 9 Wallenberg Centre for Molecular Medicine, Linköping University, Linköping, Sweden

**Keywords:** Hip, Antibiotics, Limb reconstruction, Infection control

## Abstract

**Introduction:**

Studies have shown promising results using bone graft as a carrier for local administration of antibiotics to reduce the risk of prosthetic joint infection (PJI). The objective of this clinical trial is to determine if tobramycin and vancomycin-impregnated bone graft is safe and effective in reducing the rate of PJI after total hip arthroplasty (THA).

**Methods and analysis:**

This study is an international, randomised, double-blinded, placebo-controlled clinical drug trial. Patients scheduled for THA (n=1100) requiring bone grafting (excluding revisions due to an ongoing infection) are randomised in a 1:1 ratio to prophylactic treatment with tobramycin and vancomycin or placebo-impregnated bone graft.

The primary outcome is the time to reoperation due to infection or diagnosis of PJI, expressed as a relative risk difference between the two groups. A risk reduction of at least 50% is considered clinically relevant. Secondary outcomes are time to and reason for reoperation and implant revision, type of micro-organism and antibiotic susceptibility pattern within 2 and 5 years after surgery. Safety outcomes are the number of adverse events and revision rate due to aseptic loosening. The primary analysis will be performed using proportional hazard models.

**Ethics and dissemination:**

The study has been approved under the Clinical Trial Regulation No 536/2014 (EU CT; 2024-510921-25-00). Results will be published in open-access peer-reviewed journals and disseminated to patient organisations and the media, and de-identified individual participant data will be curated and shared on reasonable request in accordance with the Findability, Accessibility, Interoperability and Reuse principles, subject to the laws and regulations governing data protection in each participating country.

**Trial registration number:**

NCT05169229.

**STRENGTHS AND LIMITATIONS OF THIS STUDY**

- This is an international, randomised, double-blinded, placebo-controlled clinical drug trial with sufficient power to determine if antibiotic-impregnated bone graft can reduce the risk of prosthetic joint infection after total hip arthroplasty.
- The pragmatic nature of the trial implies that the protocol does not dictate the surgical technique, nor the amount of bone graft used to ensure feasibility and future implementation; however, this also introduces potential performance bias between surgeons and/or sites.
- The study includes all non-infected patients requiring total hip arthroplasty with bone graft, enhancing the external validity; however, no claims can be drawn about its therapeutic use in patients with an ongoing infection.
- Tobramycin and vancomycin are widely available, inexpensive and easy to mix with bone graft and if proven effective and safe, the studied approach could offer a feasible additional treatment option to prevent prosthetic joint infections.

## Introduction

 Prosthetic joint infection (PJI) is one of the most serious complications following total hip arthroplasty (THA)^1 2^ and is associated with significant cost.^3^ PJI is the leading cause of revision surgery within 2 years with rates ranging from 0.33% to 2.45%^4 5^ after primary THA and up to 8% following aseptic revision THA.^6 7^

To prevent infection, antibiotic-loaded bone cement, combined with perioperative intravenous administration, is the standard practice in primary THA in many countries. An alternative carrier to ensure local delivery of antibiotics is morselised fresh-frozen bone graft used to fill bone defects—antibiotic-impregnated bone graft (AIBG).^8^ Adding antibiotics to fresh-frozen bone graft is a simple and cost-effective method when compared with other carriers like cement,^9^ freeze-dried bone or tricalcium phosphate.^10^ One concern regarding local antibiotics in THA is the potential selection of antibiotic-resistant bacteria. However, epidemiological data does not support this concern.^11^ It seems that the delivery medium and its associated release kinetics play an important role in the selection of antibiotic-resistant bacteria.^12^ In vitro studies have revealed prolonged release of subinhibitory antibiotic concentrations from cement,^10^ whereas AIBG results in high antibiotic levels over an extended period of time which might mitigate the development or selection of antibiotic-resistant bacteria. Moreover, drug combinations such as vancomycin and tobramycin may synergistically enhance elution kinetics, offering a promising avenue for optimising therapeutic efficacy.^13^ A combination of vancomycin and tobramycin could be effective against most bacteria found in PJI with a minimal risk of osteotoxicity^10 14^ and low systemic absorption reducing the risk of systemic toxicity.^15^ Results from smaller clinical studies are promising but a pivotal trial is needed prior to implementation of AIBG into clinical routine.^14 16^

## Methods and analysis

### Study design

**Figure 1:**
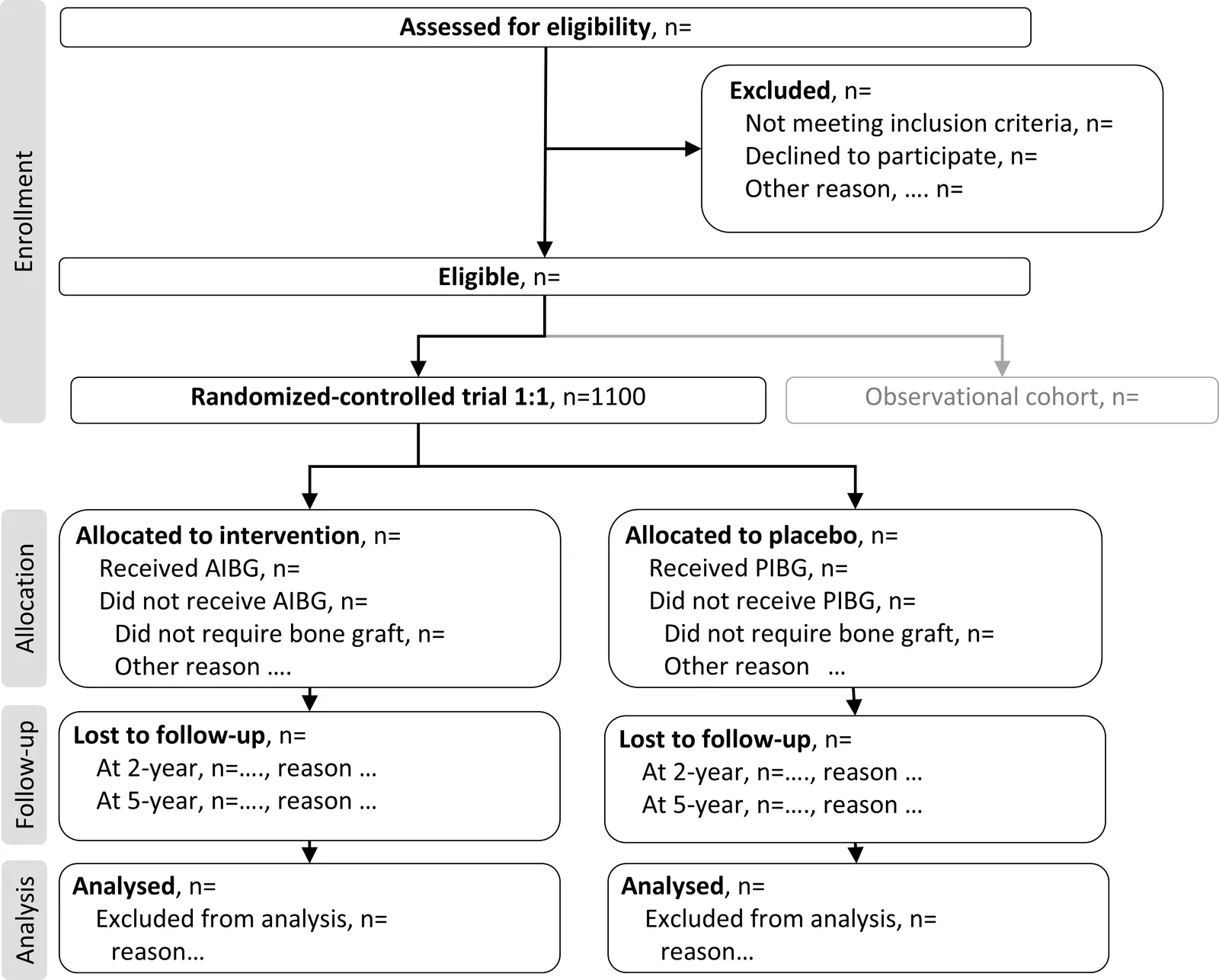
CONSORT diagram. The participants who do not consent to the randomised controlled trial are asked to participate in the parallel observational cohort. AIBG, antibiotic-impregnated bone graft; CONSORT, Consolidated Standards of Reporting Trials; PIBG, placebo-impregnated bone graft.

The ABOGRAFT trial is a phase II, international, randomised, double-blinded, placebo-controlled clinical drug trial with a total follow-up time of 5 years and a primary endpoint of PJI at 2 years postsurgery (figure 1). To date, no standard protocol for AIBG is available that could serve as the control treatment; therefore, this study is a placebo-controlled superiority trial. The study protocol is reported in accordance with the Standard Protocol Items: Recommendations for Interventional Trials (SPIRIT) statement.^17^

### Patient recruitment and withdrawal

Adults (≥18 years of age) scheduled for THA requiring bone graft are eligible for participation. Patients are recruited at orthopaedic departments by an orthopaedic surgeon based on the inclusion and exclusion criteria (table 1). Patients are informed verbally and in writing, and written informed consent is obtained prior to inclusion in the study. Patients can withdraw their consent to participate at any time point during the study (informed consent forms are available through the Clinical Trials Information System (CTIS)). All females of childbearing potential will undergo a pregnancy test on the day of surgery because both tobramycin and vancomycin are insufficiently tested for teratogenicity/fetotoxicity.

**Table 1:** Inclusion and exclusion criteria

Inclusion criteria	Exclusion criteria
Age ≥18 years Hip arthroplasty requiring bone graft Willing to provide informed consent For females of childbearing potential; a negative pregnancy test prior to surgery.	Ongoing prosthetic joint infection Known allergies and contraindications against the use of vancomycin or tobramycin Mental inability, reluctance or language difficulties that according to investigator judgement, result in difficulty understanding the meaning of study participation Expected difficulties to complete 2 year follow-up Females of childbearing potential not using contraception* Pregnant females* Nursing females*

* These are not exclusion criteria when participating in the observational cohort.

### Observational cohort

Patients will be offered participation in the observational cohort when the patient and the surgeon are unable to achieve equipoise in treatment options during their shared decision-making process, or if patients fulfil one or more of the following exclusion criteria: (1) females of childbearing potential not using contraception, (2) pregnant and (3) nursing at the time of operation. In addition, if prior to randomisation, the planned use of bone graft is not feasible, the patient will become part of the observational cohort (figure 1).

### Intervention

**Figure 2:**
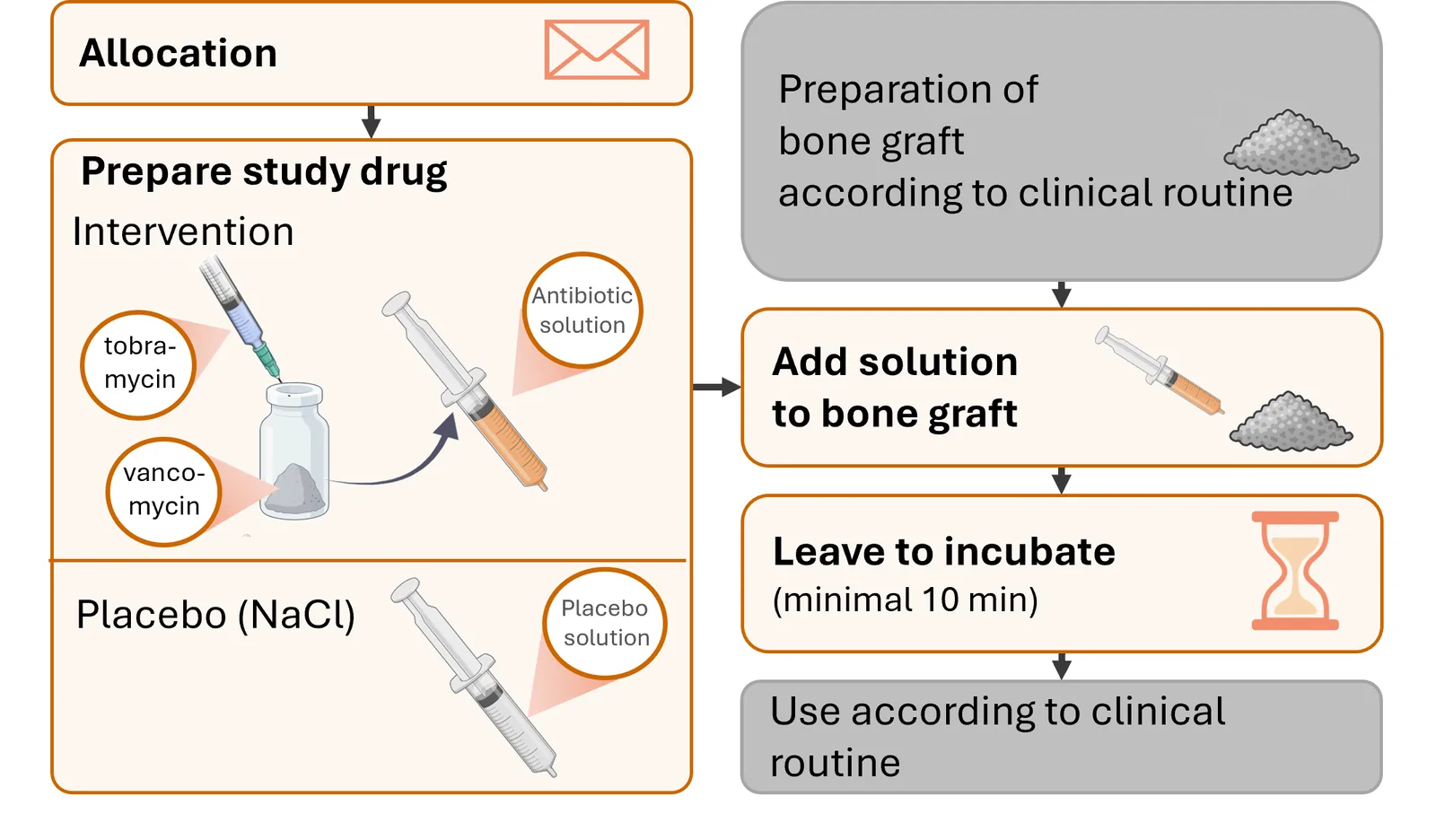
Study-specific steps. Study-specific steps are depicted in orange. Left column: 8 mL of tobramycin is used to dilute 1 g of vancomycin. Right column: after preparation, the study drug is added to the bone graft and left to incubate for 10 min. Grey boxes: procedures according to clinical routine.

The treatment drug consists of 8 mL (between 40 and 80 mg/mL=320–640 mg) tobramycin and 1 g of vancomycin.^18^ Differences in tobramycin strength are caused by differences in authorisation of tobramycin in different countries. The placebo drug consists of 8 mL of saline (NaCl 0.9%). Drug preparation is done by an unblinded trained person at the time of surgery using a closed system. The study drug is then applied onto the prepared bone graft. After mixing, the bone graft is left to incubate for a minimum of 10 min (figure 2). Most sites will use donated femoral head allografts. The recommended dose is 1 syringe (8 mL) per medium-sized femoral head allograft. Variations in the number of syringes used can be made at the surgeon’s discretion, dependent on the amount of allograft prepared.^14^ If autograft or donated bone other than femoral head is used, one syringe per approximately 30 g of prepared morselised bone graft is recommended. The maximum dose is three syringes. The bone graft is prepared according to the clinical routine established at each site. While preparation steps may vary, cleaning the marrow from the bone graft is strongly advised as this enhances the adhesion of antibiotics to the bone graft surface.^8^ All sites using allografts have a tissue establishment for handling bone registered and approved by the responsible agency in their country in accordance with the European Tissue and Cells Directive (Directive 2004/23/EC).

### Allocation and blinding

Patients are randomly assigned to either treatment or placebo in a 1:1 ratio by randomly permuted blocks of varying block size, stratified by study site by an independent monitor agency.^19^ On the day of surgery, a trained unblinded nurse (not otherwise involved in the study or treatment of the patient) performs allocation using sealed randomisation assignment envelopes. Randomisation allocation is not disclosed until 2 year follow-up is finalised for all included patients. Access to the randomisation allocation during the inclusion phase is granted only to independent monitors, the Data Monitoring Committee (DMC), and inspectors on request. Emergency code breaking for an individual patient is warranted only if the patient experiences a serious adverse event that, as judged by the treating physician, requires knowledge of the medication received.

### Study outcomes

#### Primary outcome

- Time to reoperation due to infection or diagnosis of PJI with bacteria that are susceptible to either vancomycin or tobramycin in the same hip joint within 2 years after THA.

#### Secondary outcomes

- Time to reoperation due to infection or diagnosis of PJI with any micro-organism in the same hip joint within 2 years after THA.
- Time to and cause for reoperation for any reason within 2 and 5 years.
- Time to and cause for implant revision for any reason within 2 and 5 years.
- Type of micro-organism and antimicrobial susceptibility pattern in cases with postoperative infection.

#### Safety outcomes

- Differences between the two experimental groups regarding the frequencies of adverse events.
- Differences between the two experimental groups regarding the time to aseptic loosening.

Primary and secondary outcome data will be obtained from national registers and complemented and/or validated by medical chart reviews. A diagnosis of PJI is determined based on the European Bone and Joint Infection Society definition of PJI.^20^ The type of micro-organism and antimicrobial susceptibility results from cases with an infection will be acquired through a review of medical charts and laboratory results. Patients are only included if no ongoing infection is diagnosed or suspected, however if intraoperative samples show positive cultures the type of micro-organism and antimicrobial susceptibility pattern are recorded along with the targeted postoperative antimicrobial regimen received.

Adverse events as defined by the ICH E6(R3) Good clinical practice—scientific guideline^21^ are monitored at three time points: at the day of surgery (t_0_), at discharge from the hospital (t_1_) and 2 weeks after the operation (t_2_) (table 2). Data are collected through review of the medical records and discharge documentation (t_0_ and t_1_) and through an open-ended question to the patients 2 weeks postoperatively (t_2_). As part of standard clinical practice, laboratory assessments of renal function, electrolytes and blood coagulation are performed and any deviations of the results triggering clinical interventions are recorded as adverse events. Assessment of attribution of the adverse events is done blinded by the principal investigator (an orthopaedic surgeon).

**Table 2:** Timeline and data collected during enrolment, intervention and assessment

	Enrolment		Allocation		Follow-up	
Time points	−t_1_ −1 to −365 days	t_0_ 0 days	t_1_ 1–15 days	t_2_* 14–29 days	t_3_ 2 years	t_4_ 5 years
Enrolment						
Assessment of eligibility	X					
Informed consent	X					
Randomisation		X				
Intervention						
Administration of study drug (one-time administration)		X				
Assessments						
Baseline characteristics						
Age, sex, height, weight, comorbidities, laterality, reason for operation, ASA, previous THA, if previously infected; type of micro-organism and antimicrobial susceptibility pattern	X	X				
Perioperative variables						
Blood loss, surgery time, surgical approach, fixation method, type of bone defect, type of operation, prescribed systemic antibiotics, use of additional local antibiotics, type of microbe and antimicrobial susceptibility results if micro-organisms are detected in perioperative tissue samples.		X				
Outcomes variables						
Primary outcome*:* Time to reoperation due to infection or diagnosis of PJI with bacteria that are susceptible to either vancomycin or tobramycin in the same hip joint within 2 years after THA Secondary outcome*:* Reoperation due to infection or diagnosis of PJI with any micro-organism, time to and cause for reoperation and/or implant revision for any reason after 2 and 5 years, type of micro-organism and antimicrobial susceptibility pattern in cases with postoperative infection					X	X
Safety variables†						
Adverse events		X	X	X		
Patient reported outcome measures						
Hip Dysfunction and Osteoarthritis Outcome Score 12 items and the EuroQol 5-Dimension 5-Level quality of life questionnaire	X				X	

The time frames are given as a range as these can differ due to variability between patients’ individual perioperative care and different routines at each site.

* This can coincide with the adverse event monitoring at discharge if the patient has not been discharged within 14 days.

† In addition to adverse events, the rates of revision due to aseptic loosening at 2 and 5 years are collected as safety variables. These are derived from the information collected as part of the secondary outcome; time and cause for reoperation and/or implant revision for any reason.

ASA, American Society of Anesthesiology Physical Status Classification System; PJI, prosthetic joint infection; THA, total hip arthroplasty.

Patient-Reported Outcome Measures are collected using the Hip Dysfunction and Osteoarthritis Outcome Score 12 items (HOOS-12) and the EuroQol 5-Dimension 5-Level quality of life questionnaire (EQ-5D-5L) prior to operation and 2 years postoperatively (t_3_). A 2 year follow-up period was chosen as the primary outcome because infections most often occur within this time period.^7^ Long-term follow-up is performed 5 years postoperatively (t_4_).

### Retention and adherence

To reduce selection bias, assure clinical feasibility, and improve retention and adherence, active participation of the patients after allocation is limited to the reporting of adverse events 2 weeks postoperatively, and the patient-reported outcome questionnaires (EQ-5D and HOOS-12) 2 years postoperatively. Outside the AIBG or placebo-impregnated bone graft, no additional medication is given to the patients beyond those given as part of their routine clinical care.

### Data collection

The code key, linking the study ID given to each subject’s identity, is stored at the local treatment facility protected from unauthorised access. All data will be captured in individual electronic case report forms using the Research Electronic Data Capture online platform, hosted by Linköping University. Data extraction requests from the Swedish Arthroplasty Register (SAR) or international equivalents are performed by the coordinating centre. Appropriate agreements and/or other documentation will be established in accordance with the provisions of the General Data Protection Regulation and other relevant legislation before any data transfer takes place. Source data and case report files are archived for 25 years after completion of the trial.

### Monitoring

The steering committee (SC) consists of orthopaedic surgeons and infectious disease specialists. The DMC consists of statisticians and orthopaedic surgeons. The DMC is responsible for safeguarding the interests of study participants, assessing the safety and efficacy of trial interventions, and monitoring the progress of the study. The DMC is independent of the study team and will review the primary and safety outcomes after 100 and 500 patients are included. Recommendations regarding continuation, conditionally or unconditionally, probation, termination or other modifications of the study are provided based on the cumulative review of the observed benefit or adverse effects of the treatment; as well as data observations that indicate the likelihood of definitively addressing the goals of the study. Based on the recommendations from the DMC, the SC and Sponsor will decide on alterations of study conduct, continuation, or termination of the trial. Signed charters for both the SC and the DMC are available through the CTIS. The study will be monitored by local independent monitoring organisations, coordinated by the Clinical Research Support Unit at Region Östergötland which is part of Clinical Studies Sweden. Local site monitoring is performed at regular intervals as specified in the monitoring plan. Depending on the results of the visits, this frequency can be adapted to ensure trial data integrity. The local monitor is responsible for ensuring that the trial is conducted in accordance with the approved protocol, standard operating procedures for each centre, the principles of good clinical practice and in compliance with laws and regulations.

### Patient and public involvement

A patient advisory board (PAB) was established at the start of protocol development and a mission statement for the PAB has been written to provide guidance for both the PAB and the research coordinating team. The PAB is tasked to advocate the patient’s perspective through all stages of the study. They provide input through meetings with the study coordinating team twice a year. They are informed on progress and developments and updated on SC and DMC findings. The PAB will be a crucial collaborator in the development of clinical guidelines during the implementation phase of the results of this study. Board members were recruited from the Swedish rheumatism association and the disability association in Linköping, Sweden (*Funktionsrätt Östergötland*).

### Sample size

Considering the variation in the incidence of infection across different diagnoses (eg, lower incidence in primary THA compared with revision THA), we use a weighted average incidence of PJI of 7.9%. This is based on the reported PJI incidence across diagnostic groups reported in the literature^6 22–27^ and a retrospective analysis determining the expected distribution of patients in each diagnostic group undergoing a THA using bone graft in Sweden between 2018 and 2021, as recorded in the SAR. Consistent with prior research on local antibiotic application,^28^ we assumed a 50% reduction in the incidence of PJI from 7.9% to 4.0% in the treatment group to be clinically relevant. Assuming a log HR of −0.71, derived from both risks, a total number of 62 PJIs are required at 80% power and a two-sided significance level of α=0.05. To achieve this number of complications, the smallest sample size is 1040 patients, translating to 1100 (550 in each group) when accounting for 6% attrition.

### Statistical analysis plan

All statistical analyses are based on the parallel group superiority trial design determining if the use of AIBG is superior to placebo in reducing the incidence of PJI after THA. A detailed Statistical Analysis Plan can be accessed through CTIS.

Baseline patient characteristics will be presented using descriptive statistics and reported as mean (SD) or median (range) where applicable. Analysis of the primary outcome (PJI after 2 years) will be performed when all included patients have had their 2 year follow-up. The analysis will be performed using proportional hazard models (Cox regression) to estimate the hazard ratio (with 95% CIs) between the two groups (treatment vs placebo) while adjusting for the following covariates: age, body mass index (BMI), American Society of Anesthesiology Physical Status Classification System (ASA; grouping I–II and III–IV) and relative risk for PJI (low vs high risk). Low risk for PJI refers to patients who undergo (1) first time revision for other reasons than infection, (2) THA after failed non-surgical treatment of fractures or (3) complex primary THA. High risk for PJI refers to patients who undergo (1) revision after previous PJI, (2) revision after a hip fracture, (3) re-revisions or (4) have ≥2 positive intraoperative tissue cultures with the same micro-organism. As randomisation is stratified for each site for administrative reasons, study site is added as a random effect in all models. The primary analysis will be done using the intention-to-treat principle.

Secondary outcomes related to PJI (any organism), reoperation (any reason) and revision (any reason) are analysed in a manner similar to the primary outcome. The time to the first reoperation and implant revision is estimated using the Kaplan-Meier method. Differences in cause for reoperation and implant revision are presented in tabulated form.

To determine the consistency of the treatment effect, exploratory subgroup analyses will be performed using Cox regression for the following predefined groups; age (grouped per 10 years), sex, ASA class and BMI (<30 kg/m^2^ and ≥30 kg/m^2^), and the following potential effect modifiers; study site (university or regional), preoperative diagnosis, surgical approach, type of surgery, perioperative systemic antibiotic regimen, type of micro-organism causing the infection, strength of tobramycin used and severity of bone defect.

Safety outcomes are analysed separately according to the recommendations outlined by the Consolidated Standards of Reporting Trials (CONSORT) extension to harm statement.^29^ The frequency of adverse events per participant, severity, expectedness, and duration of adverse events will be presented using descriptive statistics.

An index score measuring overall health-related quality of life will be calculated from the five dimensions of EQ-5D-5L domain scores, at baseline and after 2 years, using available value sets. A mixed model repeated measurement analysis is used for the HOOS, the EQ-5D-5L index score and the EQ-5D visual analogue scale score (EQ VAS). Censoring will be performed if a patient dies or is lost to follow-up for any reason.

All analyses will be conducted blinded to treatment allocation and according to the predefined statistical analysis plan accessible through CTIS. Statistical significance is set as a two-sided p value of 0.05 for all analyses.

### Timetable

Data collection was initiated in April 2024 and is projected to continue until December 2028 which will allow for initial analysis of the primary outcome parameter in 2030. A pilot study has been performed at Linköping University Hospital, Linköping, Sweden between 2022 and 2024.

## Discussion

### Clinical impact

The aim of this randomised controlled trial (RCT) is to determine if AIBG is safe and effective in reducing the rate of PJI after THA. As high-quality randomised clinical trials on the prophylactic effect of AIBG are lacking,^14 16^ large practical variation exists in the utilisation of AIBG and the method of preparation and administration of AIBG between healthcare providers. PJI significantly affects patient health and mortality while also imposing substantial financial burden on healthcare systems and society as a whole.^1 3^ Therefore, investigating cost-effective and practical preventive strategies to lower PJI rate is of utmost importance.^2^ This will be the first RCT with sufficient power and long-term follow-up, to determine whether prophylactic use of AIBG is an effective and safe treatment option alongside current perioperative prophylactic antibiotic regimens. This study will also serve as the foundation for the development of clinical guidelines for the safe application of AIBG as an infection prophylaxis in THA.

### Strengths and limitations

This study is pragmatic to ensure the external validity of the results and conclusions. To further increase feasibility and limit reliance on patient retention, we chose our outcome parameters based on data availability in the national arthroplasty registers complemented and validated with medical record data.

While no study method can reduce all biases, the choice of a randomised, double-blinded and placebo-controlled design reduces potential selection, treatment and allocation biases. This RCT design is challenging due to the large number of required participating patients. We have mitigated this challenge by first ensuring that the inclusion criteria are broad and all currently non-infected patients requiring THA with bone graft can be included. Group differences related to the indication for THA are included as a covariate based on the relative risk for PJI allowing for generalisation to the practical reality of a diverse patient cohort. Second, this study does not delineate how the surgeon uses bone graft. While this approach might increase the risk of performance bias between surgeons and centres, it ensures generalisability to a broad spectrum of clinical practices. Moreover, based on clinical practice and the patient’s needs, different types (eg, allograft vs autograft) and amounts of bone graft will be used, therefore the quantity of local antibiotics administered will differ between patients.

The authorised strength of tobramycin varies between countries, ranging from 40 mg/mL to 80 mg/mL. While vancomycin is the antibiotic expected to target the large majority of micro-organisms responsible for PJI encountered^30^ differences in the strength of tobramycin used might influence the results. As such tobramycin strength is added as a potential effect modifier in the subgroup analysis. Simultaneously, in vitro microbiological studies are performed to understand the variability in antibiotic effect when altering tobramycin strength.

This study focuses on the potential benefits and risks of AIBG as a prophylaxis in patients undergoing THA due to any reason other than infection. As such, no conclusions can be drawn regarding its efficacy when used as a treatment for already infected prosthetic joints. In summary, this randomised, double-blinded clinical drug trial can provide crucial information about the efficacy and long-term risks of AIBG as a prophylactic treatment option against PJI after THA.

### Ethics and dissemination

The study has been approved under the Clinical Trial Regulation No 536/2014 (EU CT; 2024-510921-25-00) and is registered at ClinicalTrials.gov (NCT05169229). We will facilitate data sharing in line with the Findability, Accessibility, Interoperability and Reuse principles, considering European laws and guidelines. Study results will be reported in accordance with the CONSORT statement^31^ and its relevant extensions.^29^ The results will be submitted for publication in open-access peer-reviewed academic journals and disseminated to patient organisations and the media.
