# Supplementary material for: Antibiotic-impregnated bone graft to prevent infection after total hip arthroplasty (ABOGRAFT): protocol for a randomised, double-blind, placebo-controlled trial
Source: BMJ Open. 2026 Sep 11;16(9):e114161. doi: 10.1136/bmjopen-2025-114161 (PMC13583874; doi:10.1136/bmjopen-2025-114161)
Supplement: Supplementary file 1 [file bmjopen-2025-114161.reviewer_comments.pdf]

PEER REVIEW HISTORY

BMJ Open publishes all reviews undertaken for accepted manuscripts. Reviewers are asked to complete a checklist review form and are provided with free text boxes to elaborate on their assessment. These free text comments are reproduced below.

ARTICLE DETAILS

Title (Provisional)

Antibiotic-impregnated bone graft to prevent infection after total hip arthroplasty (ABOGRAFT): Protocol for a randomised, double-blind, placebo-controlled trial

Authors

Wezenberg, Daphne; Hailer , Nils P; Sköldenberg, Olof; Stefánsdóttir, Anna; Tsikandylakis, Georgios; Wildeman, Peter; Kärrholm, Johan; Söderquist, Bo; Nilsson, Lennart E; Schilcher, Jörg

---

VERSION 1 - REVIEW

**Reviewer**	**1**
**Name**	**Fokter, Samo**
**Affiliation**	**University Clinical Centre, Maribor, Slovenia**
**Date**	**19-Mar-2026**
**COI**	**None**

I commend the authors for their thoughtful and well-structured study protocol entitled "Antibiotic-Impregnated Bone Graft to Prevent Infection After Total Hip Arthroplasty (ABOGRAFT): Protocol for a Randomised, Double-Blind, Placebo-Controlled Trial." This study aims to evaluate whether bone graft impregnated with tobramycin and vancomycin is safe and effective in reducing the incidence of prosthetic joint infection (PJI) following total hip arthroplasty (THA).

The manuscript is logically organized and clearly written. The Introduction provides relevant background and appropriately highlights the clinical importance of PJI as a leading cause of early revision after primary and revision THA. The Methods and Analysis section thoroughly describes the study design, inclusion criteria, intervention, and statistical framework. The inclusion of informative tables and figures further enhances clarity and transparency. Study limitations are adequately acknowledged at the end of the Discussion section.

Overall Recommendation

This is a well-prepared and clinically relevant study protocol addressing an important and timely question in arthroplasty. The methodology is robust, and the study design is

appropriate to answer the proposed research question. I recommend acceptance in its current form.

**Reviewer**	**2**
**Name**	**Gould, Daniel**
**Affiliation**	**The University of Melbourne, Department of Surgery**
**Date**	**03-May-2026**
**COI**	**None**

I thank the editorial team for the opportunity to review this manuscript.

It details the design of a large clinical trial that is clearly the result of years of thorough planning, with careful consideration given to all aspects of the trial.

The clinical problem being addressed is meaningful, and this trial has the potential to generate new evidence that will guide changes in practice to improve patient outcomes.

I have some suggestions to improve the manuscript prior to recommending acceptance for publication:

- Suggest explaining why the authors did not use the MSIS criteria to define PJI (Parvizi J, Tan TL, Goswami K, Higuera C, Della Valle C, Chen AF, Shohat N. The 2018 Definition of Periprosthetic Hip and Knee Infection: An Evidence-Based and Validated Criteria. J Arthroplasty. 2018 May;33(5):1309-1314.e2. doi: 10.1016/j.arth.2018.02.078. Epub 2018 Feb 26. PMID: 29551303.). I understand that this is a European clinical trial, therefore using the EBJIS definition is reasonable, however some readers may expect the MSIS criteria to be used and a pre-emptive explanation may be beneficial to allay any concerns.

- Please expand upon the criteria for selection of covariates (ASA, BMI, etc.) in the Cox regression model? What was the process for selecting these variables? Were any other considered?

- - Furthermore, please expand upon the way in which the 'PJI risk' variable was defined. The authors have clearly outlined the defining characteristics for high and low risk, however it is unclear how these criteria were selected. Are there any references which can be provided to support this decision/definition?

- Suggest collecting reasons why surgeon/patient felt equipoise was deemed to not be reached. This may not be feasible, but something to consider. A qualitative analysis of these data could inform further research into the concerns of patients and clinicians regarding when/when not to use graft, to determine whether these concerns are valid.

VERSION 1 - AUTHOR RESPONSE

Reviewer: 1
Dr. Samo Fokter, University Clinical Centre, Maribor, Slovenia
Comments to the Author:
I commend the authors for their thoughtful and well-structured study protocol entitled "Antibiotic-Impregnated Bone Graft to Prevent Infection After Total Hip **Arthroplasty** (ABOGRAFT): Protocol for a Randomised, Double-Blind, Placebo-Controlled Trial." This study aims to evaluate whether bone graft impregnated with tobramycin and vancomycin is safe and effective in reducing the incidence of prosthetic joint infection (PJI) following total hip arthroplasty (THA).
The manuscript is logically organized and clearly written. The Introduction provides relevant background and appropriately highlights the clinical importance of PJI as a leading cause of early revision after primary and revision THA. The Methods and Analysis section thoroughly describes the study design, inclusion criteria, intervention, and statistical framework. The inclusion of informative tables and figures further enhances clarity and transparency. Study limitations are adequately acknowledged at the end of the Discussion section.
Overall Recommendation
This is a well-prepared and clinically relevant study protocol addressing an important and timely question in arthroplasty. The methodology is robust, and the study design is appropriate to answer the proposed research question. I recommend acceptance in its current form.
Response: Thank you for your comments.

Reviewer: 2
Mr. Daniel Gould, The University of Melbourne
Comments to the Author:
I thank the editorial team for the opportunity to review this manuscript.
It details the design of a large clinical trial that is clearly the result of years of thorough planning, with careful consideration given to all aspects of the trial.
The clinical problem being addressed is meaningful, and this trial has the potential to generate new evidence that will guide changes in practice to improve patient outcomes.

I have some suggestions to improve the manuscript prior to recommending acceptance for publication:
- Suggest explaining why the authors did not use the MSIS criteria to define PJI (Parvizi J, Tan TL, Goswami K, Higuera C, Della Valle C, Chen AF, Shohat N. The 2018 Definition of Periprosthetic Hip and Knee Infection: An Evidence-Based and Validated Criteria. J Arthroplasty. 2018 May;33(5):1309-1314.e2. doi: 10.1016/j.arth.2018.02.078. Epub 2018 Feb 26. PMID: 29551303.). I understand that this is a European clinical trial, therefore using the EBJIS definition is reasonable, however some readers may expect the MSIS criteria to be used and a pre-emptive explanation may be beneficial to allay any concerns.
Our response: We thank the reviewer for raising this point. We are aware of the ongoing work to harmonize the MSIS and EBJIS criteria, however currently the MSIS criteria are not yet endorsed by the European Bone and Joint Infection Society (EBJIS). We have therefore designed the trial to record individual diagnostic markers. This allows the Statistical Analysis Plan to be adapted as the definition of PJI continues to evolve over the coming years.
Suggested change: No change to the manuscript.

- Please expand upon the criteria for selection of covariates (ASA, BMI, etc.) in the Cox regression model? What was the process for selecting these variables? Were any other considered?

Our response: *The covariates were pre-specified as established baseline risk factors for PJI rather than chosen by data-driven selection, consistent with guidance on covariate adjustment in randomised trials (EMA 2015; ICH E9) and with the approach taken in comparable arthroplasty trials, which adjust for similar patient-level factors (e.g. Abedi et al., Pro-Hip-Quality, BMJ Open 2023). We adjust for age, BMI, ASA classification, and the dichotomous PJI-risk stratum (further elaborated on below), each established risk factors for PJI and routinely recorded at every participating site, which also ensures completeness and feasibility. Study site is included as a random effect because randomization is stratified by site. Additional potential interactive variables (for example, causative organism) are not used for adjustment but are examined in pre-specified exploratory subgroup analyses. The complete list of variables of sensitivity and subgroup analysis is provided in the Statistical Analysis Plan.*

Suggested change: *No change to the manuscript; the full covariate list is specified in the Statistical Analysis Plan.*

- Furthermore, please expand upon the way in which the 'PJI risk' variable was defined. The authors have clearly outlined the defining characteristics for high and low risk, however it is unclear how these criteria were selected. Are there any references which can be provided to support this decision/definition?

Our response: *The dichotomous PJI-risk variable was introduced to account for baseline heterogeneity in infection susceptibility across the study population. The classification follows established literature on patient groups at increased risk of PJI, and we have added supporting references in the manuscript and below. The high-risk group was defined as follows: Patients undergoing arthroplasty after hip fracture(Zhao, Parel et al. 2024), revision procedures after PJI(Goumenos, Hardt et al. 2024, Joanroy, Gubbels et al. 2024), or re-revision procedures(Badarudeen, Shu et al. 2017, Cascales, Foissey et al. 2026) as these indications are consistently associated with a greater risk of subsequent PJI. In addition, the presence of 2 or more positive intraoperative microbiological samples with the same microorganism was considered a marker of increased infection risk (Hipfl, Mooij et al. 2021, Neufeld, Lanting et al. 2021, Schwarze, Moellenbeck et al. 2023). Because the trial includes a clinically heterogeneous population, this stratification adjusts for important baseline differences in predisposition to PJI related to the indication for THA and to the microbiological findings.*

Suggested change: *Supporting references for the high-risk indications and for positive intraoperative cultures were added at the definition of the PJI-risk variable, and to the reference list.*

Location: *Statistical analysis, definition of the relative risk for PJI (low versus high).*

- Suggest collecting reasons why surgeon/patient felt equipoise was deemed to not be reached. This may not be feasible, but something to consider. A qualitative analysis of these data could inform further research into the concerns of patients and clinicians regarding when/when not to use graft, to determine whether these concerns are valid.

Our response: *We thank the reviewer for this suggestion and agree that understanding why equipoise is not achieved is valuable. During screening, we document the reason each screened patient is excluded, including cases in which the surgeon or the patient is unwilling to proceed. These entries capture the situations in which equipoise is not reached, as well as the interpretations of exclusions and inclusion criteria, especially regarding the amount of bone graft used and the sometimes difficult assessment of whether the patient is considered*

infection-free. We plan to summarize these descriptively, grouped into categories. This assessment will provide structured, qualitative insight into the decision-making process, including equipoise.
Importantly, patients who are eligible but decline randomization, or found not to need sufficient amounts of bone graft perioperatively, are followed in separate observational cohort.
Suggested change: No change to the manuscript; the reasons for exclusion recorded during screening may be explored qualitatively in future work.

References

Badarudeen, S., A. C. Shu, K. L. Ong, D. Baykal, E. Lau and A. L. Malkani (2017). "Complications After Revision Total Hip Arthroplasty in the Medicare Population." Journal of Arthroplasty **32**(6): 1954-1958.

Cascales, V., C. Foissey, M. Mercier, R. Coulomb, M.-H. Fessy, S. Lustig and P. Kouyoumdjian (2026). "Why revision of total hip arthroplasty fails: a retrospective consecutive cohort study of 963 patients." Bone & Joint Open **7**(2): 148-157.

Goumenos, S., S. Hardt, V. Kontogeorgakos, A. Trampuz, C. Perka and S. Meller (2024). "Success Rate After 2-Stage Spacer-Free Total Hip Arthroplasty Exchange and Risk Factors for Reinfection: A Prospective Cohort Study of 187 Patients." The Journal of Arthroplasty **39**(10): 2600-2606.

Hipfl, C., W. Mooij, C. Perka, S. Hardt and G. I. Wassilew (2021). "Unexpected low-grade infections in revision hip arthroplasty for aseptic loosening." The Bone & Joint Journal **103-B**(6): 1070-1077.

Joanroy, R., S. Gubbels, J. K. Møller, S. Overgaard and C. Varnum (2024). "Risk of second revision and mortality following first-time revision due to prosthetic joint infection after primary total hip arthroplasty: results on 1,669 patients from the Danish Hip Arthroplasty Register." Acta Orthopaedica **95**.

Neufeld, M. E., B. A. Lanting, M. Shehata, J. L. Howard, S. J. MacDonald, M. G. Teeter and E. M. Vasarhelyi (2021). "Prevalence and Outcomes of Unexpected Positive Intraoperative Cultures in Presumed Aseptic Revision Hip Arthroplasty." JBJS **103**(15): 1392-1401.

Schwarze, J., B. Moellenbeck, G. Gosheger, J. Puetzler, N. Deventer, T. Kalisch, K. N. Schneider, S. Klingebiel and C. Theil (2023). "The Role of Single Positive Cultures in Presumed Aseptic Total Hip and Knee Revision Surgery-A Systematic Review of the Literature." Diagnostics (Basel) **13**(9).

Sebastian, S., J. Parvizi and J. G. Hofstaetter (2025). "Diagnostic Guidelines for Periprosthetic Joint Infection: Know the Differences." The Journal of Arthroplasty **40**(8): 1933-1935.

Sigmund, I. K., M. Luger, R. Windhager and M. A. McNally (2022). "Diagnosing periprosthetic joint infections." Bone & Joint Research **11**(9): 608-618.

Sotiriou, D., H. Stigbrand and G. Ullmark (2020). "Is two-stage revision surgery for infected hip arthroplasty worth the suffering, resources and results compared to one-stage?" Hip Int.

Sousa, R., A. Ribau, P. Alfaro, M.-A. Burch, J. Ploegmakers, M. McNally, M. Clauss, M. Wouthuyzen-Bakker and A. Soriano (2023). "The European Bone and Joint Infection Society definition of periprosthetic joint infection is meaningful in clinical practice: a multicentric validation study with comparison with previous definitions." Acta Orthopaedica **94**: 8-18.

Streck, L. E., C. M. Sterneder, L. Haralambiev, M. Brenneis, Y.-F. Chiu and F. Boettner (2025). "Significant differences in the rate of periprosthetic joint infections in revision hip and knee arthroplasty depending on the applied definition." Archives of Orthopaedic and Trauma Surgery **145**(1).

Zhao, A. Y., P. M. Parel, A. R. Agarwal, A. Gu, R. A. Ranson, A. Das, G. J. Golladay and S. C. Thakkar (2024). "Increased Risk of 10-Year Revision Following Total Hip Arthroplasty for Femoral Neck Fracture." The Journal of Arthroplasty.

VERSION 2 - REVIEW

Reviewer 2

Name Gould, Daniel

Affiliation The University of Melbourne, Department of Surgery

Date 24-Jun-2026

COI

All of my comments have been addressed, and I have no further suggestions.

VERSION 2 - AUTHOR RESPONSE

Reviewer: 2

Mr. Daniel Gould, The University of Melbourne

Comments to the Author:

All of my comments have been addressed, and I have no further suggestions.

<u>Our response:</u> We thank the reviewer for their time and effort invested in this manuscript.
